# Tafazzin catalyzes transacylation and hydrolysis with inverse acyl specificities

**DOI:** 10.1016/j.jlr.2026.101169

**Published:** 2026-10-08

**Authors:** Michael Schlame, Yang Xu, Mindong Ren, Julio Lopes Sampaio, Daniel Levy

**Affiliations:** 1 Department of Anesthesiology, New York University Langone Medical Center, New York, NY, USA; 2 Institut Curie, Research Center, Paris, France

**Keywords:** cardiolipin, enzymology/enzyme mechanism, fatty acid remodeling, glycerophospholipids, lipolysis and fatty acid metabolism, mitochondria

## Abstract

Tafazzin is a phospholipid-lysophospholipid acyltransferase that remodels fatty acids of mitochondrial phospholipids. To study the tafazzin mechanism, we expressed and purified the enzyme, measured its kinetics with different pairs of phosphatidylcholine (PC) and lysophosphatidylcholine (LPC) species, and simulated the reactions in a mathematical model based on the mass-action law. Variations in chain length and PC:LPC ratio showed that tafazzin reacts with micelles but not with bilayers. In the presence of micellar substrates (LPC + short-chain PC), transacylation rates measured at different substrate ratios suggested similar affinities for PC and LPC. Surprisingly, tafazzin catalyzed both transacylation and hydrolysis of fatty acids, the former preferring oleoyl over palmitoyl and the latter preferring palmitoyl over oleoyl groups. To study the remodeling of cardiolipin, we measured the reaction of trilinoleoyl-monolyso-cardiolipin with palmitoyl-oleoyl-PC and simulated it in a mathematical model. Simulations predicted and measurements confirmed a large number of bidirectional transacylations between the initial substrates, between substrates and products, and between different products. This led to a gradual diversification of the lipid composition from 2 to more than 15 species. Discrepancies between observed and simulated compositions demonstrated transacylation preference for oleoyl over palmitoyl groups. The data are consistent with a bidirectional ping-pong mechanism that operates through a single low-affinity binding site and suggest that transacylation specificity can at least in part be explained by selective loss of fatty acids through hydrolysis.

Cardiolipin (CL) is a mitochondrial phospholipid that strongly interacts with proteins of oxidative phosphorylation and that is necessary for optimal performance of mitochondria 1, 2. The metabolic stability of CL and its protein association critically depend on unsaturated fatty acids 3, 4 that are incorporated into CL by acyl remodeling 5, 6. CL remodeling is accomplished by the combined activities of a lipase and an acyltransferase, expressed by *Abhd18* 7, 8 and *Tafazzin* 9, 10, respectively.

Between the two enzymes, tafazzin has been studied more extensively because mutations in *Tafazzin* cause Barth syndrome, a mitochondrial disease presenting with cardiomyopathy, skeletal muscle weakness, fatigue, and neutropenia (11). Ablation of *Tafazzin* from experimental models, such as yeast, flies, mice, and human-derived organoids, consistently lowers respiratory activity, respiratory coupling, and ATP-dependent functions 12, 13, 14, 15. The data demonstrate that fatty acid remodeling of CL is important for energy metabolism. We have shown that remodeling protects CL from degradation and suggested that the underlying mechanism is an increase in protein association, which makes CL inaccessible to lipases 4, 16.

We have shown that tafazzin is a phospholipid-lysophospholipid acyltransferase (10) that can transfer various kinds of fatty acids between different lipid classes (17). The tertiary structure of tafazzin was resolved by cryo-EM and found to be largely consistent with the AlphaFold prediction (18). Further improvements in image quality identified the amino acids of the catalytic site and revealed non-covalently attached acyl-CoA near the active center (19). The data suggest a ping-pong enzymatic mechanism, by which tafazzin moves fatty acids from phospholipids to protein-bound CoA and from CoA to lysophospholipids (Fig. 1A).

**Fig. 1:**
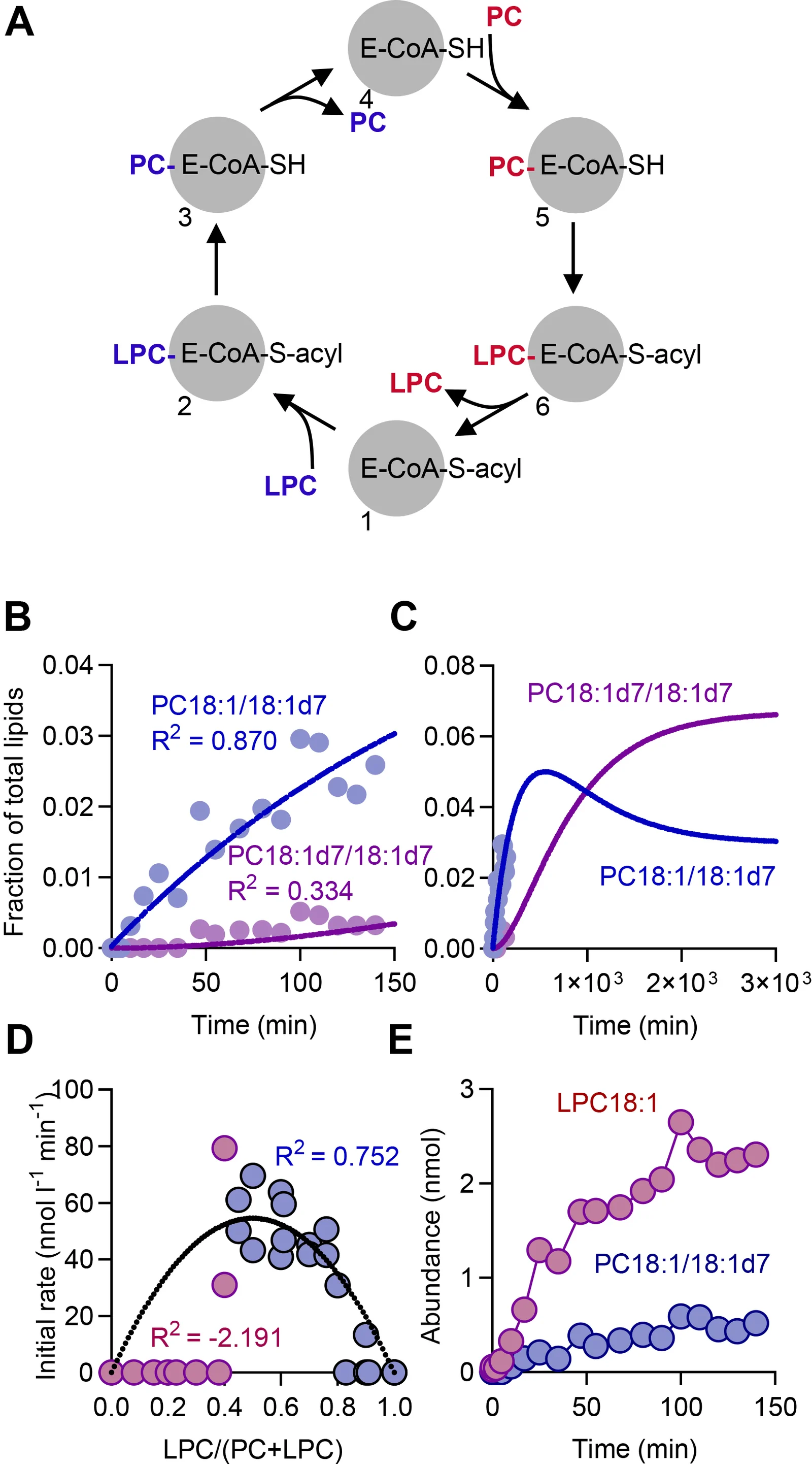
Tafazzin reshuffles labeled oleoyl groups between PC and LPC. Purified tafazzin (1 μM) was incubated with dioleoyl-PC (PC18:1/18:1) and deuterium-labeled oleoyl-LPC (LPC18:1d7) at a total lipid concentration of 1 mM. Incubations were carried out in a volume of 20 μl and products were measured by mass spectrometry. A: The reaction is depicted as a ping-pong mechanism operating with CoA as a prosthetic group bound to tafazzin (E). Different colors indicate different molecular species of PC and LPC. B and C: Measurements were made after different reaction times at a PC:LPC ratio of 1:9. Solid lines show mathematical simulations. The goodness of fit was evaluated by the coefficient of determination (*R*^2^). D: Measurements were made after 40 min of incubation at different PC:LPC ratios. Solid line shows mathematical simulation. The goodness of fit (coefficient of determination, *R*^2^) was evaluated separately for LPC > PC and for LPC < PC. E: Data show the concentrations of LPC18:1 and PC18:1/18:1d7 after different reaction times at a PC:LPC ratio of 1:9. , lysophosphatidylcholine; PC, phosphatidylcholineLPC.

Despite progress in elucidating the catalytic mechanism, several questions remain unanswered, all having important physiologic implications. (i) The reaction of tafazzin is reversible 17, 20 yet it is not known whether tafazzin actually catalyzes bidirectional transacylations in the chemical equilibrium state and whether that is necessary for CL remodeling. If tafazzin does indeed operate in forward and reverse direction, it is expected to both acylate monolyso-cardiolipin (MLCL) and de-acylate CL. In that case it is not clear why lipase activity, in addition to tafazzin, is necessary for CL remodeling 13, 21, 22. (ii) In vivo, tafazzin forms CL species with unsaturated fatty acids, yet in vitro, tafazzin is a promiscuous acyltransferase that reacts with virtually all types of fatty acids and all phospholipid classes 17, 20. The underlying cause of acyl specificity has been controversial. Either the phase state of lipids 17, 20 or the substrate specificity of tafazzin 23, 24 has been offered as explanation. (iii) Finally, in view of previous publications 17, 20, 23, 24, it remains to be confirmed whether tafazzin activity is indeed conditional on specific packing states of lipids.

To explore equilibrium states, acyl specificities, and phase dependence, we made kinetic measurements of a simplified reaction between phosphatidylcholine (PC) and lysophosphatidylcholine (LPC), in which we modified physical properties of substrates solely by varying length and saturation of the acyl chains. We then explored more complex transacylations between PC and MLCL. Analysis by mass spectrometry enabled us to record in parallel multiple reaction products and to compare their progression to predictions made by mathematical simulations.

## Materials and methods

### Expression and purification of tafazzin

Tafazzin from *Saccharomyces cerevisiae* was expressed and purified as described before with minor modifications (20). The open reading frame of the *S. cerevisiae* gene encoding tafazzin (TAZ1) was amplified by the polymerase chain reaction and inserted into pGEX-EF, a GST-fusion protein expression vector. BL21 E. coli cells were transformed with pGEX-EF-TAZ1 plasmid DNA. Cells were grown to the early logarithmic phase (OD600 = 0.6) in 1.25 L Luria-Bertani broth and protein expression was induced by adding 1 mM isopropyl 1-thio-β-D-galactopyranoside. Cells were allowed to express the recombinant protein at 32°C for 3.5 h, after which they were harvested by centrifugation and stored frozen at −80°C. Frozen cell pellets (total wet weight of 12 g) were resuspended in a total volume of 30 ml ice-cold PBS containing 1 mM EDTA, 100 μg/ml lysozyme, 10 mM β-mercaptoethanol, 0.1% Triton X-100, and protease inhibitor cocktail (Sigma-Aldrich). Cells were broken up with a high-pressure homogenizer. Debris and unbroken cells were removed by centrifugation at 10,000 x g for 30 min at 4°C. The supernatant was incubated for 2 h with 8 ml bed volumes of Glutathione Sepharose 4B resin (Santa Cruz Biotechnology). Tubes were slowly rotated at 4°C. The resin was removed by centrifugation at 500 x g for 5 min and washed 3 times with cold PBS containing 1 mM EDTA and protease inhibitor cocktail. The recombinant protein was eluted from the washed resin in 2.5 ml elution buffer, containing 50 mM Tris (pH8.0), 1 mM EDTA, and 10 mM glutathione. Eluted protein was concentrated with a 10 kDa filter and applied to a Sephadex 200 column for size exclusion chromatography to separate free GST from the recombinant protein. Fractions containing the recombinant protein were pooled and assessed by SDS-PAGE. The protein concentrations of the final enzyme preparations ranged from 1 to 2 g/l. Tafazzin was inactivated by incubating the purified protein in a boiling water bath for 3 min.

### PC-LPC transacylation assays

Lipids were dried at 40°C under a stream of nitrogen and resuspended in aqueous reaction buffer (10 mM Tris, pH 7.5) by sonication in a water bath. Reactions were performed at 35°C in 40 μl buffer, containing lipids at a total concentration of 2 mM and tafazzin at a concentration of 10 μM. Reactions were stopped after the specified time intervals by adding 0.4 ml 2-propanol/acetonitrile (3:2). This solution was mixed 1:1 with matrix solution containing 9-aminoacridine (20 g/l) in 2-propanol/acetonitrile (3:2) and applied to a target plate for matrix-assisted-laser-desorption-ionization time-of-flight mass spectrometry. Spectra were acquired in positive ion mode on a Bruker Autoflex instrument (Bruker Daltonics) operated in reflectron mode. Data from 1000 acceptable laser shots (resolution >2500) were collected in increments of 50 shots per laser position. The laser position was changed randomly. We demonstrated that PC and LPC yielded equal signal intensities per molar concentration. In order to quantify fatty acids released from phospholipids, we analyzed lipid extracts by liquid-chromatography electrospray-ionization mass spectrometry exactly as described (7). To measure ion current intensities of the carboxylate anions of specific fatty acids, spectra were imported into Xcalibur 4.1.50 and analyzed with the Quality Browser.

### PC-MLCL transacylation assays

We incubated 64 nmol 1-palmitoyl-2-oleoyl-PC (PC16:0/18:1) and 64 nmol trilinoleoyl-MLCL (MLCL18:2/18:2/18:2) with or without 572 nmol galactosyl-ceramide (GalCer). All lipids were obtained from Avanti Polar Lipids. Premixed lipids were evaporated in a vacuum for 2 h and resuspended in 0.1 ml buffer containing 20 mM Hepes (pH 7.2) and 200 mM NaCl. Reactions were initiated by adding 1.3 nmol tafazzin (final concentration: 10 μM). The mixture was stirred with a magnetic bar for up to 45 min at 30°C. Aliquots of 20 μl were withdrawn at different times for lipid analysis. Lipids were extracted with chloroform-methanol. The organic phase was collected and evaporated in a SpeedVac. Extracts were dissolved in 100 μl solvent consisting of propanol/chloroform/methanol (4/1/2, by vol) and 7.5 mM ammonium acetate. Samples were analyzed by direct infusion into a QExactive Plus mass spectrometer (Thermo Fisher Scientific) equipped with a TriVersa NanoMate ion source (Advion Biosciences). Data were acquired in centroid mode and analyzed with the lipid identification software LipidXplorer-1.2.8.1 (25). Tolerance was set to 2 ppm.

### Mathematical simulation of transacylations

We simulated the tafazzin reaction by assuming (i) random transacylations between all potential pairs of donor and acceptor species and (ii) equal affinity for all molecular species. First, we built a complete list of theoretical products from two substrates by rearranging the fatty acids in every possible way. Next, we compiled an inventory of all possible transacylations between acyl donors (D_i_, i = 1, 2, …, m) and acyl acceptors (A_j_, j = 1, 2, …, n) on the list. Finally, we calculated the rate of each reaction using the chemical rate law:

(1) $$v_{ij}{=kd}_{i}a_{j\;}$$

In this relation, *v*_*ij*_ represents the velocity of acyl transfer from D_i_ to A_j_, *k* is the uniform rate constant, and *d*_*i*_ and *a*_*j*_ are the concentrations of D_i_ and A_j_ respectively, both expressed as fractions of the total number of lipid molecules:

(2) $$\sum_{i}^{m}d_{i}+\sum_{j}^{n}a_{j}=1$$

Simulations were performed in incremental steps over fixed time intervals. In each iteration, product concentrations were calculated from substrate concentrations by equation system (1) and then used as substrate concentrations for the next iteration. We typically performed several hundred to a few thousand iterations to simulate the evolution of a reaction.

For the simulation of PC-LPC transacylations, we considered positional isomers, which yielded a list of 8 product species. For example, when PC8:0/8:0 reacts with LPC13:0, 4 PC species and 4 LPC species can be formed. This accounts for 16 donor-acceptor pairs, each allowing 2 alternative transacylations, yielding a total of 32 reactions (Table 1).

**Table 1:** The table lists all possible PC-LPC transacylations in a reaction starting with 1,2-dioctanoyl-phosphatidylcholine (PC8:0/8:0) and 1-tridecanoyl-2-lyso-phosphatidylcholine (LPC13:0)

Reaction	PC Substrate		LPC Substrate		LPC Product		PC Product	
	Sn-1	Sn-2	Sn-1	Sn-2	Sn-1	Sn-2	Sn-1	Sn-2
1	8:0	8:0	13:0	None	8:0	None	13:0	8:0
2	8:0	8:0	13:0	None	None	8:0	13:0	8:0
3	8:0	8:0	None	13:0	8:0	None	8:0	13:0
4	8:0	8:0	None	13:0	None	8:0	8:0	13:0
5	8:0	8:0	8:0	None	8:0	None	8:0	8:0
6	8:0	8:0	8:0	None	None	8:0	8:0	8:0
7	8:0	8:0	None	8:0	8:0	None	8:0	8:0
8	8:0	8:0	None	8:0	None	8:0	8:0	8:0
9	8:0	13:0	13:0	None	8:0	None	13:0	13:0
10	8:0	13:0	13:0	None	None	13:0	13:0	8:0
11	8:0	13:0	None	13:0	8:0	None	13:0	13:0
12	8:0	13:0	None	13:0	None	13:0	8:0	13:0
13	8:0	13:0	8:0	None	8:0	None	8:0	13:0
14	8:0	13:0	8:0	None	None	13:0	8:0	8:0
15	8:0	13:0	None	8:0	8:0	None	13:0	8:0
16	8:0	13:0	None	8:0	None	13:0	8:0	8:0
17	13:0	8:0	13:0	None	13:0	None	13:0	8:0
18	13:0	8:0	13:0	None	None	8:0	13:0	13:0
19	13:0	8:0	None	13:0	13:0	None	8:0	13:0
20	13:0	8:0	None	13:0	None	8:0	13:0	13:0
21	13:0	8:0	8:0	None	13:0	None	8:0	8:0
22	13:0	8:0	8:0	None	None	8:0	8:0	13:0
23	13:0	8:0	None	8:0	13:0	None	8:0	8:0
24	13:0	8:0	None	8:0	None	8:0	13:0	8:0
25	13:0	13:0	13:0	None	13:0	None	13:0	13:0
26	13:0	13:0	13:0	None	None	13:0	13:0	13:0
27	13:0	13:0	None	13:0	13:0	None	13:0	13:0
28	13:0	13:0	None	13:0	None	13:0	13:0	13:0
29	13:0	13:0	8:0	None	13:0	None	8:0	13:0
30	13:0	13:0	8:0	None	None	13:0	8:0	13:0
31	13:0	13:0	None	8:0	13:0	None	13:0	8:0
32	13:0	13:0	None	8:0	None	13:0	13:0	8:0

For the simulation of PC-MLCL transacylations, we did not consider positional isomers in order to reduce complexity. Consequently, the product list contained 3 LPC species, 6 PC species, 10 MLCL species, and 15 CL species (Table 2), which accounts for 21 acyl donors and 13 acyl acceptors, yielding 273 distinct donor-acceptor pairs. Each PC species can donate either the fatty acid in sn-1 position or the fatty acid in sn-2 position while each CL species has 4 different choices. Altogether, 936 different reactions were included in the equation system.

**Table 2:** The table lists all molecular species that can be formed when tafazzin reacts with 1-palmitoyl-2-oleoyl-PC (PC16:0/18:1) and trilinoleoyl-MLCL (MLCL18:2/18:2/18:2)

Class	Number	Species
LPC	1	LPC16:0
	2	LPC18:1
	3	LPC18:2
PC	1	PC16:0/16:0
	2	PC16:0/18:1
	3	PC16:0/18:2
	4	PC18:1/18:1
	5	PC18:1/18:2
	6	PC18:2/18:2
MLCL	1	MLCL16:0/16:0/16:0
	2	MLCL16:0/16:0/18:1
	3	MLCL16:0/18:1/18:1
	4	MLCL18:1/18:1/18:1
	5	MLCL18:1/18:1/18:2
	6	MLCL18:1/18:2/18:2
	7	MLCL18:2/18:2/18:2
	8	MLCL16:0/16:0/18:2
	9	MLCL16:0/18:2/18:2
	10	MLCL16:0/18:1_18:2
CL	1	CL16:0/16:0/16:0/16:0
	2	CL16:0/16:0/16:0/18:1
	3	CL16:0/16:0/18:1/18:1
	4	CL16:0/18:1/18:1/18:1
	5	CL18:1/18:1/18:1/18:1
	6	CL16:0/16:0/16:0/18:2
	7	CL16:0/16:0/18:2/18:2
	8	CL16:0/18:2/18:2/18:2
	9	CL18:2/18:2/18:2/18:2
	10	CL18:1/18:1/18:1/18:2
	11	CL18:1/18:1/18:2/18:2
	12	CL18:1/18:2/18:2/18:2
	13	CL16:0/18:1/18:2/18:2
	14	CL16:0/18:1/18:1/18:2
	15	CL16:0/16:0/18:1/18:2

## Results

### Tafazzin reshuffles fatty acids in the equilibrium state

To measure the tafazzin reaction in the chemical equilibrium state, we incubated the purified, recombinant enzyme with dioleoyl-phosphatidylcholine (PC18:1/18:1) and deuterium-labeled 1-oleoyl-2-lyso-phosphatidylcholine . Transacylations between these two substrates alter the distribution of label but not the chemical composition because products, like substrates, are oleoyl species of PC and LPC, respectively. At a PC:LPC ratio of 1:9 where lipids are organized in mixed micelles, we observed the gradual accumulation of PC with one or two deuterium-labeled chains (Fig. 1B). The formation of double-labeled PC requires at least two transacylations, in which an unlabeled chain is first moved from PC to labeled LPC and then a labeled chain is moved back from single-labeled PC to labeled LPC. Thus, labeled and unlabeled oleoyl groups were transferred back and forth between isotopomers, demonstrating bidirectional transacylations in the equilibrium state.

We simulated this reaction in a kinetic model, in which we assumed equal affinities for PC and LPC. Simulations matched the relative rates by which single-labeled and double-labeled PC formed in the experiment. In particular, single-labeled PC rose much faster than double-labeled PC at the beginning of the reaction even though double-labeled PC is predicted to become more abundant at the end of the reaction because >80% of oleoyl groups were labeled (Fig. 1C). When varying the PC and LPC concentrations, simulations predicted the reaction rate to reach its maximum at a PC:LPC ratio of 1:1. Experiments confirmed this prediction but, in contrast to the simulations, showed complete loss of activity when the abundance of PC exceeded that of LPC (Fig. 1D). The data show that tafazzin activity increases when donor and acceptor approach equal concentrations but vanishes when substrates transition from the micellar to the lamellar phase. The latter confirms previous results from our lab 17, 20.

In summary, tafazzin catalyzed transacylations in the equilibrium state with similar affinities for PC and LPC provided that substrates are in a micellar phase state.

### Tafazzin catalyzes transacylation and hydrolysis

When we analyzed the stoichiometry of the oleoyl exchange between PC and LPC, we noticed that unlabeled LPC formed faster than labeled PC (Fig. 1E). This discrepancy suggested that oleoyl groups were released by hydrolysis. To investigate this phenomenon further, we examined a series of PC-LPC substrates carrying different acyl chains.

First, we incubated tafazzin with dioctanoyl-PC (PC8:0/8:0) and tridecanoyl-LPC (LPC13:0), two lipids that remain in the micellar state at all PC:LPC ratios 26, 27, 28. In this case, transacylation rates followed the substrate ratio dependence predicted by mathematical simulations not only for PC < LPC but also for PC > LPC, peaking at a ratio of unity. Likewise, the relative rates at which primary (PC8:0/13:0) and secondary (PC13:0/13:0) transacylation products formed, agreed with the simulations (Fig. 2A). This supports the model’s assumption of equal affinities for all PC and LPC species as long as lipids remain in the micellar phase state. However, just like in the previous experiment, LPC8:0 increased faster than PC8:0/13:0, suggesting that PC-LPC transacylation was accompanied by PC hydrolysis (Fig. 2B).

**Fig. 2:**
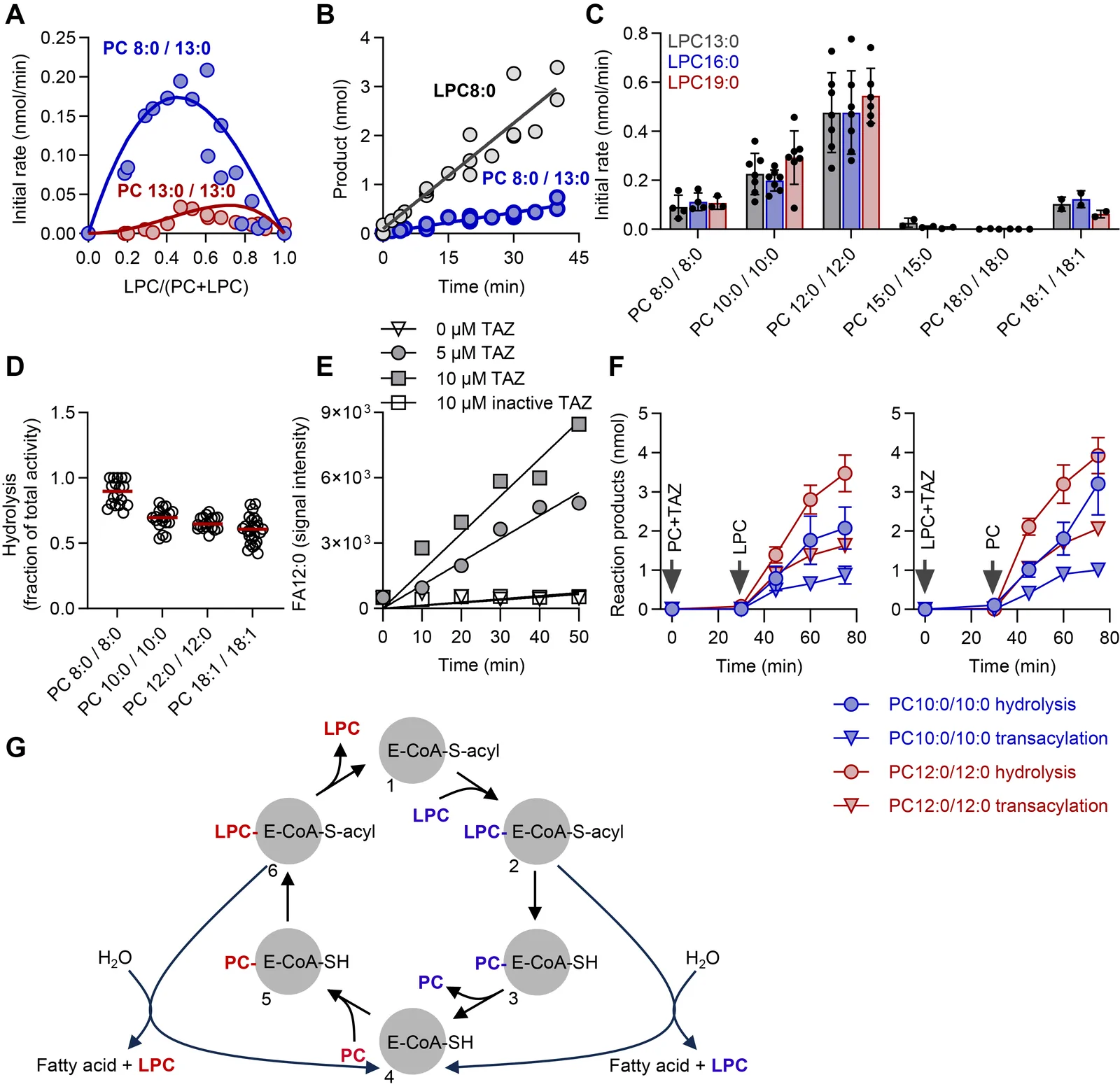
Tafazzin catalyzes transacylation and hydrolysis. A and B: Purified tafazzin (10 μM) was incubated with dioctanoyl-PC (PC8:0/8:0) and tridecanoyl-LPC (LPC13:0) at a total lipid concentration of 1 mM. Reaction products were measured by mass spectrometry. Incubation time was 30 min in panel A and the PC:LPC ratio was 1:9 in panel B. Solid lines in panel a represent mathematical simulations (*R*^2^ = 0.7). Solid lines in panel B represent linear regression (*R*^2^ = 0.9). C: Tafazzin (10 μM) was incubated with the indicated PC and LPC species (1 mM each). Initial transacylation rates were determined by measurements at 35 min. Columns show means and SDs (N = 3–6). D: Proportion of hydrolysis to transacylation was determined by mass spectrometry (reaction conditions as in C). Individual measurements are shown with mean values. E: Tafazzin (TAZ, different concentrations) was incubated with 1 mM dilauroyl-PC (PC12:0/12:0) and 1 mM palmitoyl-LPC (LPC16:0). Free lauric acid (FA12:0) was quantified by mass spectrometry. Heat-inactivated tafazzin was included as a control. F: Tafazzin (10 μM) was preincubated either with 1 mM PC (species as indicated) or with 1 mM LPC16:0. After 30 min, the second substrate (1 mM) was added, and incubation was continued. Transacylation and hydrolysis were measured by mass spectrometry. Data are means with standard errors (N = 3). G: Cartoon shows how tafazzin may release free fatty acids. E-CoA-S-acyl (intermediate 1) represents the ground state of the enzyme. Exogenous LPC initiates the catalytic cycle by removing the enzyme-bound acyl group. Exogenous PC is hydrolyzed by release of fatty acid from LPC-E-CoA-S-acyl (intermediate 6). LPC, lysophosphatidylcholine; PC, phosphatidylcholine.

Next, we gradually increased the length of acyl chains. Raising the chain length of PC from 8 to 12 carbon atoms increased transacylation activity whereas raising the LPC chain length had no effect. However, further rise in PC chain length to 15 or 18 carbon atoms, which puts PC into the gel state at reaction temperature (28), caused a drastic reduction in activity. This was partially reversed by adding a double bond, which restored fluidity (Fig. 2C). Stoichiometric analysis of the products showed that hydrolysis occurred with all substrates and that the hydrolysis to transacylation ratio was not much affected by the chain length (Fig. 2D). Importantly, we confirmed by mass spectrometry that free fatty acids were released commensurate with reaction time and enzyme activity (Fig. 2E).

To elucidate the mechanism of hydrolysis, we preincubated tafazzin with PC before adding LPC and then repeated the experiment in reverse order. We found that neither transacylation nor hydrolysis occurred before the second substrate was added, regardless of the sequence (Fig. 2F). The fact that LPC was required not only for PC-LPC transacylation, where it is expected, but also for PC hydrolysis, demonstrates that both reactions proceed through an LPC-dependent catalytic intermediate, such as LPC-E-CoA-S-acyl (Fig. 2G). The idea that fatty acids are released from LPC-E-CoA-S-acyl is also supported by the fact that E-CoA-S-acyl, the only other intermediate with a hydrolyzable S-acyl group, is stable enough to survive protein purification (19).

In summary, tafazzin catalyzed PC-LPC transacylation and PC hydrolysis. Catalytic efficiency of both activities increased with the length of acyl chains until the changing physical properties triggered transition into the bilayer phase state. Transacylation rates at different PC:LPC ratios under phase-invariant conditions, suggest that PC and LPC react with similar affinities.

### Tafazzin catalyzes forward and reverse transacylations between multiple species of PC, MLCL, LPC, and CL

Next, we replaced the acyl acceptor LPC with MLCL. Transacylations from PC to MLCL are the presumed biological function of tafazzin 29, 30 but it is difficult to measure such reactions in vitro because PC and MLCL form bilayers that do not react with tafazzin (17). Accordingly, only 2.7 ± 0.3% (N = 3) of lipids reacted within 45 min when purified tafazzin was incubated with vesicles consisting of 1-palmitoyl-2-oleoyl-PC (PC16:0/18:1) and trilinoleoyl-MLCL (MLCL18:2/18:2/18:2). However, the transacylation activity was stimulated more than 10-fold (35.1 ± 8.4% of lipids, N = 3) when we added GalCer (GalCer/PC/MLCL = 9/1/1, by mol).

The mechanism by which GalCer stimulated transacylations was not investigated here. However, it is known that GalCer forms nanotubes 31, 32, 33 because its molecules are tilted and chiral 34, 35, which is expected to create a packing conflict with non-tilted lipids like PC and MLCL. This packing conflict could disorganize phospholipids and make them more accessible to tafazzin but other mechanisms, such as changes in membrane fluidity or membrane architecture, could also explain the GalCer effect.

In the presence of GalCer, tafazzin transferred acyl groups from PC to MLCL, producing LPC and CL. This reaction was accurately simulated by our mathematical model assuming random transacylations with equal affinities (Fig. 3A). Since we were primarily interested in the formation of CL and its molecular species, we limited our analysis to transacylations and did not include hydrolysis in the mathematical model, nor did we quantify the release of fatty acids during the reaction.

**Fig. 3:**
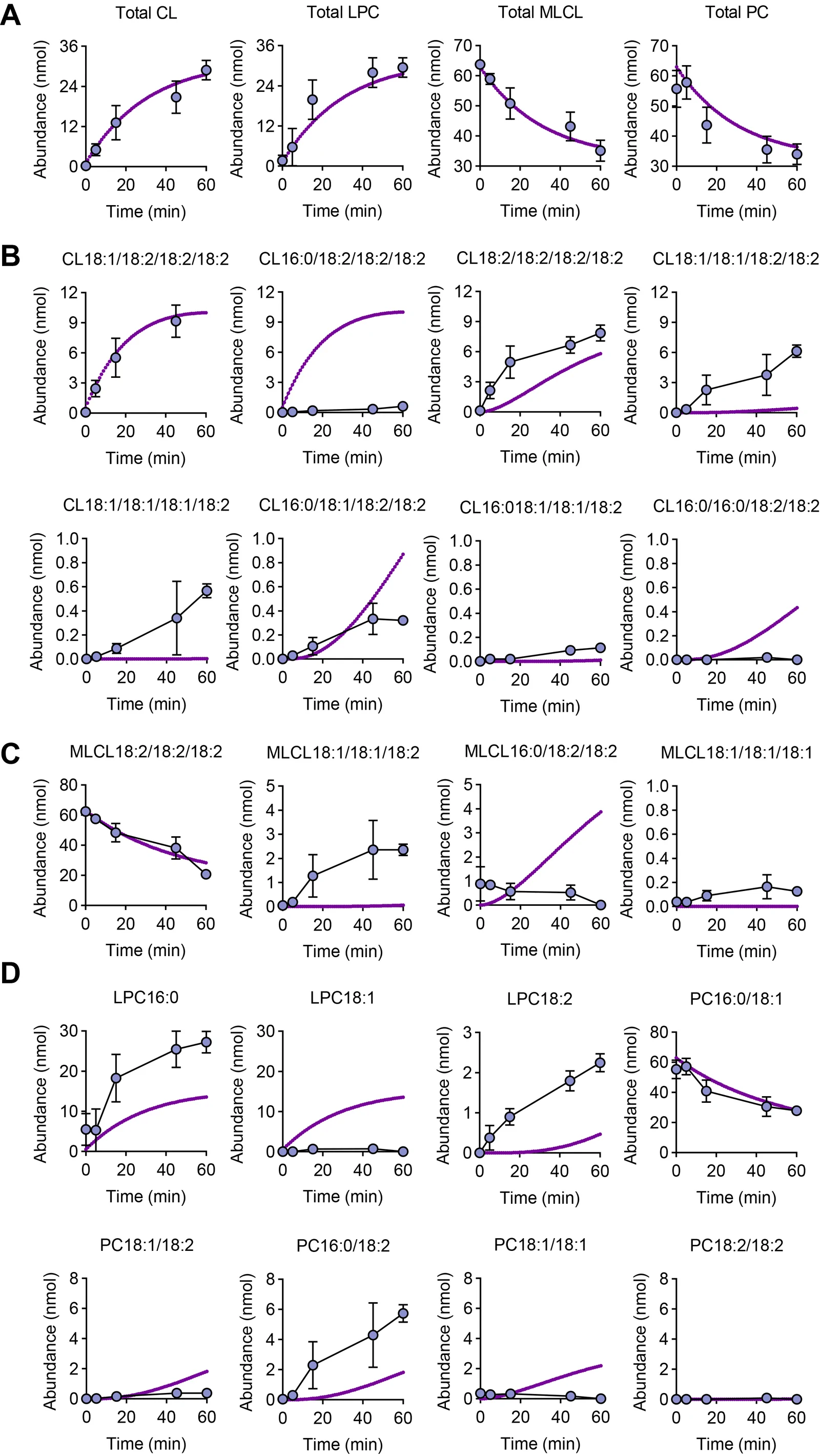
Tafazzin generates multiple molecular species from two substrates. Tafazzin (10 μM) was incubated with PC16:0/18:1 (0.5 mM), MLCL(18:2)_3_ (0.5 mM), and galactosylceramide (4.3 mM). The lipid composition was measured by mass spectrometry (blue circles) and simulated in a mathematical model (purple lines). Data show the evolution of the lipid class composition (A) and the evolution of different molecular species of CL (B), MLCL (C), LPC and PC (D). Measured data are means with standard error (N = 3). CL, cardiolipin; LPC, lysophosphatidylcholine; MLCL, monolyso-cardiolipin; PC, phosphatidylcholine.

The mathematical model predicted, and experiments confirmed, multiple competing reactions not only between the initial substrates but also between products and substrates and between different products, which created an increasingly complex species composition. For instance, we observed a steady rise of CL(18:2)_4_, which requires acyl transfer from PC16:0/18:1 to MLCL18:2/18:2/18:2 followed by linoleoyl transfer from newly formed CL16:0/18:2/18:2/18:2 or CL18:1/18:2/18:2/18:2 to MLCL18:2/18:2/18:2. We also observed species like CL(18:1)_3_18:2 and CL16:0(18:1)_2_18:2, which can only be produced by 5 or more consecutive transacylations (Fig. 3B).

While experiment and model agreed that net conversion of lipid classes (PC + MLCL → LPC + CL) was associated with the emergence of multiple new species, caused by parallel transacylations from PC to MLCL, from PC to LPC, from CL to MLCL, and from CL to LPC, their quantities were accurately predicted only for some but not all molecular species. For example, CL18:1(18:2)_3_ formed as predicted by the model but CLs containing palmitoyl groups were less abundant than predicted and other CLs containing oleoyl and linoleoyl groups were more abundant than predicted (Fig. 3B). Similar discrepancies were observed among MLCL species (Fig. 3C). LPC16:0 formed much faster than LPC18:1, confirming that more oleoyl groups than palmitoyl groups were transferred from PC to MLCL. The higher abundance of LPC16:0 compared to LPC18:1 explains why the formation of PC16:0/18:2 was preferred over the formation of PC18:1/18:2 (Fig. 3D).

In summary, incubation of tafazzin with PC16:0/18:1 and MLCL(18:2)_3_ created >15 new molecular species through a complex system of transacylations among multiple donor-acceptor pairs. Mathematical simulation assuming random transacylations with uniform affinity, predicted correctly the net conversion of PC + MLCL into LPC + CL but not the compositional changes observed within each lipid class. Hence, acyl groups were transferred selectively, in particular, oleoyl groups were transferred more efficiently than palmitoyl groups from PC to MLCL.

### Acyl hydrolysis provides transacylation specificity

To determine whether the preference for oleoyl over palmitoyl groups was due to their carbon position, we compared 1-palmitoyl-2-oleoyl-PC with 1-oleoyl-2-palmitoyl-PC. We found strong preference for oleoyl transfer from PC to MLCL regardless of the isomer, excluding the carbon position as the source of oleoyl specificity. However, oleoyl transfer from 1-palmitoyl-2-oleoyl-PC was faster than oleoyl transfer from 1-oleoyl-2-palmitoyl-PC, suggesting higher reactivity of acyl groups at the sn-2 position (Fig. 4A).

**Fig. 4:**
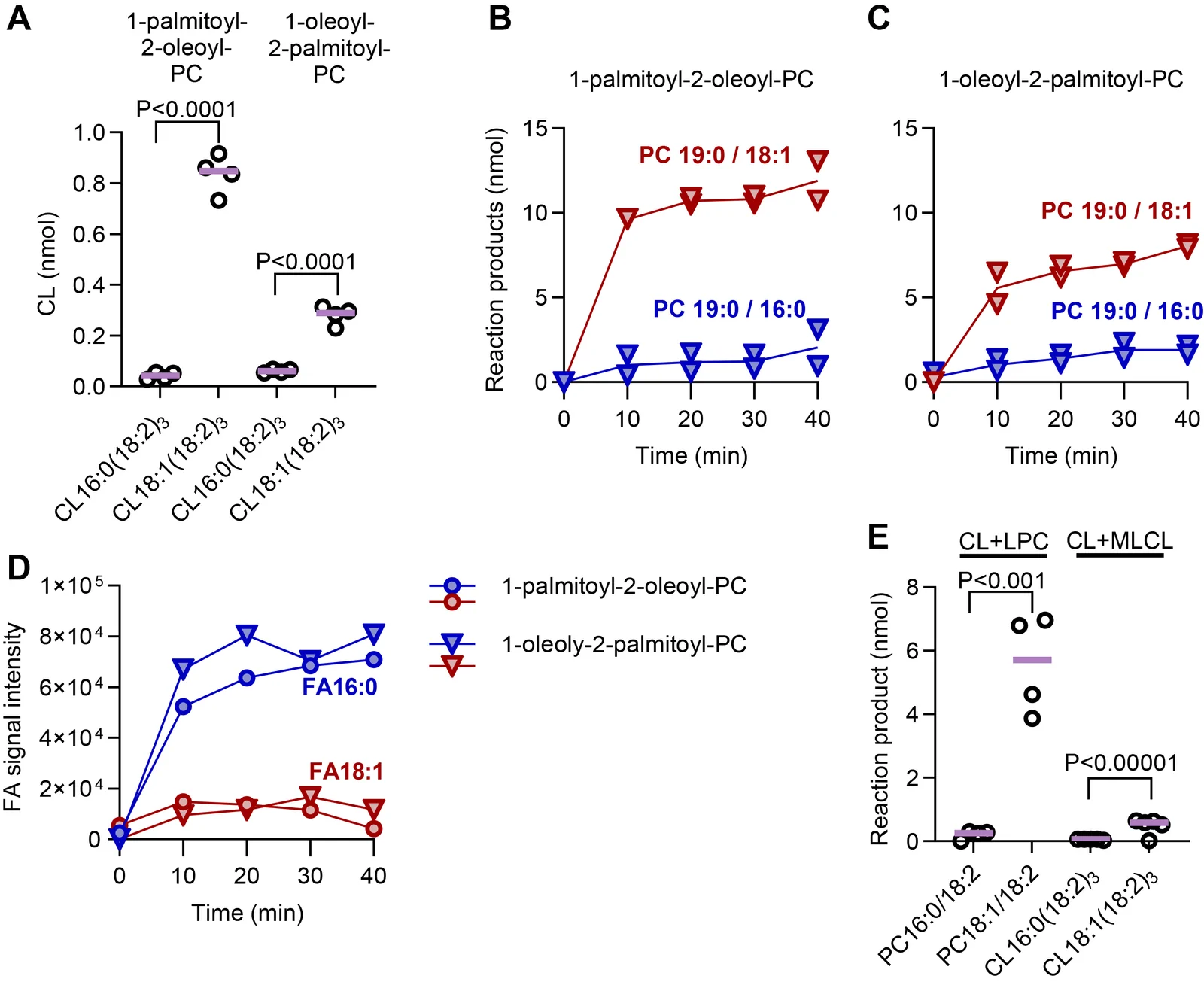
Transacyation and hydrolysis have inverse acyl specificity. A: Tafazzin (10 μM) was incubated with galactosylceramide (4.3 mM), MLCL(18:2)_3_ (0.5 mM), and different positional isomers of PC16:0/18:1 (0.5 mM) for 15 min. Cardiolipin was measured by mass spectrometry. Mean values were compared by *t* test. B, C, and D: Tafazzin (2 μM) was incubated with LPC19:0 (0.5 mM) and different positional isomers of PC16:0/18:1 (0.125 mM). PC products and fatty acids (FA) were measured by mass spectrometry. E: Tafazzin (2 μM) was incubated with CL(16:0/18:1)_2_ (0.5 mM), CaCl_2_ (20 mM), and either LPC18:2 (0.125 mM) or MLCL(18:2)_3_ (0.5 mM) for 20 min. PC and CL species were measured by mass spectrometry. Mean values were compared by *t* test. LPC, lysophosphatidylcholine; MLCL, monolyso-cardiolipin; PC, phosphatidylcholine.

To validate this conclusion, we measured the reaction of the two PC isomers with LPC19:0. Again, more oleoyl groups than palmitoyl groups were transferred regardless of whether the acyl donor was 1-palmitoyl-2-oleoyl-PC (Fig. 4B) or 1-oleoyl-2-palmitoyl-PC (Fig. 4C). The data demonstrate the preference for oleic acid over palmitic acid in PC-LPC transacylations. However, the opposite was true for the accompanying hydrolysis reaction. Free palmitic acid was released much faster than free oleic acid from both PC isomers, showing that hydrolysis and transacylation had inverse acyl specificities (Fig. 4D). Finally, we measured competition between palmitoyl and oleoyl groups in transacylations from CL to LPC and from CL to MLCL. In both reactions, strong preference for oleic acid confirmed the general validity of the transacylation specificity (Fig. 4E).

In summary, tafazzin transferred oleoyl groups more efficiently than palmitoyl groups from different donors to different acceptors. The acyl specificity of transacylation was the inverse to the acyl specificity of hydrolysis, suggesting that selective hydrolysis of palmitic acid promotes oleoyl transacylation.

## Discussion

We discovered that tafazzin releases fatty acids in addition to transferring them from phospholipids to lysophospholipids. Dual lipase-transacylase activities have been described for lipases, but they are mechanistically different from tafazzin. Lipases either release acyl groups from an acyl-enzyme intermediate (hydrolase activity) or pass them on to an acceptor (transacylase activity). Typically, the two reactions are competitive, and the more acceptor is present the less hydrolysis occurs 36, 37, 38. In contrast, tafazzin requires the acceptor for both transacylation and hydrolysis, implying that the mechanism of hydrolysis relies on an acceptor-dependent transition state of the enzyme.

Kinetic analysis of tafazzin is confounded by a number of physical effects. Changes in lipid concentration and composition may affect the phase state of lipid particles (e.g. micelles or bilayers), the rate of substrate diffusion both within and between particles, and the association of particles with the enzyme. All may influence the kinetic behavior of tafazzin. We carried out experiments at high enzyme:substrate ratios in order to minimize the need for interparticle diffusion. Regardless, experiments remain susceptible to shifts in phase state and enzyme association, which precludes the application of canonical kinetic measurements, in which the two substrate concentrations are varied independently. Instead, we made kinetic measurements at variable substrate ratios but constant total substrate concentrations. These measurements matched mathematical simulations where equal affinities were presumed for the two substrates. Our data show that the maximal reaction velocity occurs at a donor-to-acceptor ratio of 50:50 while half-maximal velocity occurs at donor-to-acceptor ratios of 15:85 and 85:15, respectively. Thus, a lipid concentration of about 15% for either donor or acceptor, is the best estimate of a “K_m_-value” we can derive from our data. Although this result was obtained in artificial micelles and may not strictly match the kinetic behavior of tafazzin in mitochondrial membranes, it suggests that affinities are low, which implies that effective transacylation requires high concentrations of both the donor and the acceptor. This may explain why the remodeling of CL is dependent on a lipase, such as CLD1 or ABHD18, that can supply sufficient amounts of MLCL to tafazzin.

*In vivo*, tafazzin enriches unsaturated fatty acids in CL 29, 30. No consensus has been reached about the origin of acyl specificity, which could either be caused by the self-organization of lipids (thermodynamic model) or by the enzymatic specificity of tafazzin (kinetic model). We have shown that transacylation rates of different substrates correlate with the amount of product in the equilibrium state, which suggests that rates are controlled by the energy difference between the initial and the final lipid composition (20). The idea that physical properties of lipids determine specificity has also been supported by effects of the phase state on specificity (17) and by the promiscuous usage of fatty acids by human tafazzin expressed in genetically distant hosts 39, 40. Alternatively, tafazzin may carry an acyl-specific binding site that selects unsaturated fatty acids 23, 24. This idea was born out of data showing strong acyl specificity of PC-MLCL transacylations with various molecular species. However, when comparing substrates with different acyl carbons, double bond numbers, double bond positions, or cis-trans configurations in the presence of high detergent concentrations, concomitant effects on fluidity and acceptor-donor miscibility confound interpretation of the data.

Here we show that selective hydrolysis of fatty acids supports transacylation specificity. By preferentially hydrolyzing palmitic rather than oleic acid, more oleoyl than palmitoyl groups may be transferred from the donor to the acceptor. Consistent with that, we found strong preference for oleoyl over palmitoyl groups in transacylations from PC to MLCL, from CL to LPC, from PC to LPC, and from CL to MLCL. Despite enzymatic specificity, present and published experiments show that tafazzin can hydrolyze and transacylate a range of saturated and unsaturated fatty acids 10, 17, 20, 23, 24. Because tafazzin can both transfer and hydrolyze different fatty acids, it may generate a spectrum of lipid compositions. If meaningful energy differences exist between these compositions, the ones with lower energy will prevail regardless of enzymatic specificities.

Our data confirm that bilayer perturbations are necessary for CL remodeling in mitochondrial membranes. We reported that tafazzin requires its substrates to be in a non-bilayer state 17, 20 but this conclusion was challenged by the finding that transacylation takes place between bilayer lipids like PC and MLCL 23, 24. However, in latter studies, more than 40 percent of lipids were detergent molecules, which drastically raises fluidity and compromises the bilayer state (41). Here we altered the phase state by incrementally changing the PC:LPC ratio in the absence of detergents. We showed that transacylation activity collapses as soon as PC makes up more than 60 percent of lipids unless the acyl chains are short enough to maintain the micellar phase state. This demonstrates unequivocally that tafazzin does not react with lipids in the bilayer state. *In vivo*, the bilayer state is perturbed by the insertion of large amounts of proteins (42), which alters the physical properties of mitochondrial lipids (43). Since lipids have to be pulled out of the membrane to reach the catalytic site 18, 19, the energy barrier for lipid recruitment depends on how well lipids are integrated into the membrane (44). This may explain why lipids of well-packed bilayers that are stabilized by favorable lipid-lipid interactions, are inaccessible to tafazzin whereas lipids of protein-rich bilayers are accessible to tafazzin.

In conclusion, we have shown that tafazzin catalyzes hydrolysis in addition to transacylation. Hydrolysis supports the acyl specificity of transacylation by selectively removing saturated fatty acids. Purified tafazzin can remodel CL in vitro by a complex exchange of fatty acids between multiple lipid species due to its ability to catalyze reversible transacylations. Our data are consistent with a ping-pong mechanism of tafazzin that operates with a single lipid binding site.

## Data availability

All data are contained within the article.

## Conflict of interest

The authors declare that they have no conflicts of interest with the contents of this article.
